# Task representation in individual and joint settings

**DOI:** 10.3389/fnhum.2015.00268

**Published:** 2015-05-12

**Authors:** Wolfgang Prinz

**Affiliations:** Department of Psychology, Max Planck Institute for Human Cognitive and Brain SciencesLeipzig, Germany

**Keywords:** task, event codes, response conflict, agent conflict, interference, task sharing

## Abstract

This paper outlines a framework for task representation and discusses applications to interference tasks in individual and joint settings. The framework is derived from the Theory of Event Coding (TEC). This theory regards task sets as transient assemblies of event codes in which stimulus and response codes interact and shape each other in particular ways. On the one hand, stimulus and response codes compete with each other within their respective subsets (horizontal interactions). On the other hand, stimulus and response code cooperate with each other (vertical interactions). Code interactions instantiating competition and cooperation apply to two time scales: on-line performance (i.e., doing the task) and off-line implementation (i.e., setting the task). Interference arises when stimulus and response codes overlap in features that are irrelevant for stimulus identification, but relevant for response selection. To resolve this dilemma, the feature profiles of event codes may become restructured in various ways. The framework is applied to three kinds of interference paradigms. Special emphasis is given to joint settings where tasks are shared between two participants. Major conclusions derived from these applications include: (1) Response competition is the chief driver of interference. Likewise, different modes of response competition give rise to different patterns of interference; (2) The type of features in which stimulus and response codes overlap is also a crucial factor. Different types of such features give likewise rise to different patterns of interference; and (3) Task sets for joint settings conflate intraindividual conflicts between responses (what), with interindividual conflicts between responding agents (whom). Features of response codes may, therefore, not only address responses, but also responding agents (both physically and socially).

## Tasks as Targets of Research

Experimental research in cognition and action relies on the study of performance in controlled environmental settings. *Reaction time* *tasks* provide a typical example of the way in which this research is conducted. The mentioned tasks require participants to perform certain actions in response to certain events, thereby following certain rules they have been instructed to follow (or, in somewhat more technical terms, to select and perform speeded *responses* to upcoming *stimuli* according to pre-specified *mapping rules*). Task performance is assessed in terms of reaction time, that is, the time that elapses between stimulus presentation and response onset. Observed relationships between experimental conditions and task performance then lay the ground for theory building. Theories specify representational architectures and functional processing mechanisms that may underlie (and hence explain) the empirical observations reflecting these relationships.

In this approach tasks are tools, not targets of research. Tasks are understood as means for providing the information that theory requires, without being studied as ends in themselves. For instance, to run a memory experiment it is taken for granted that participants understand the instructions and implement an appropriate *task set*, that is a cognitive representation of the task, and that subserves both implementing the task and performing it. Researchers take the operation of task sets for granted in the same way that tailors take the workings of scissors and sewing machines for granted. Just as tailors use these tools for making garments and robes, researchers use tasks as tools for building theories. These theories address cognitive architectures and mechanisms, but not the tasks used to probe them. As long as the tools that researchers use are instrumental for attaining the desired targets, they can afford to be ignorant about their technical functionality.

While it is, on the one hand, perfectly justified to use tasks as *tools for* theory-driven research, it is, on the other hand, certainly no less legitimate to address tasks as *targets of* theory-driven research. This is what the present paper aims at. Importantly, theories of task representation and control will not only shed light on the workings of research tools in experimental settings, but also elucidate a central feature of human performance, viz. the interaction between bottom-up and top-down control of cognition and action. Tasks require us to pursue goals and perform goal-directed actions under appropriate circumstances. Thus, in a sense, we may see ourselves engaging in tasks all the time in everyday life. From this perspective research tools for experimental settings may be seen to instantiate a constituitive feature of action control in more natural settings. Understanding one will therefore translate into understanding the other.

More than a century ago Ach (1905) pioneered an approach to the representational underpinnings of task control. In his studies of simple arithmetic tasks the main focus was the issue of how a task’s goal (e.g., *to* *multiply* two digits) is represented besides the material objects to which it applies (i.e., *the* *digits* presented). In his theoretical account he introduced the famous notion of *determining tendencies* that were meant to instantiate intended goals, on top of *reproductive tendencies* meant to instantiate represented objects. Ach’s analysis was based on both, subjective experience (i.e., introspection) and objective performance (i.e., measurements). In the meantime reliance on subjective experience has, for a number of good reasons, become much weaker than reliance on objective performance. One of the classical instruments for objective assessment of task performance is provided by reaction time tasks, on which I will focus here. Typical issues that come to mind when we raise these tasks from tools to targets of enquiry include questions such as: How can we characterize task sets? How can they be acquired either through learning or verbal instruction? Which kinds of representational resources does their implementation require? How are stimuli and responses represented? How can mapping rules be instantiated in task sets? What does it take to move from representation to control, that is, from understanding a task to carrying it out?

The last two decades have witnessed the emergence of a wealth of literature on issues of cognitive control in reaction time tasks (Kiesel et al., 2010; Koch et al., 2010; Logan et al., 2014; Verbruggen et al., 2014). The main bulk of this literature is based on paradigms for the study of flexibility in task representation and control (e.g., in tool use, task switching and stop signal paradigms). Accordingly, the theoretical focus is on short-term changes within and between task sets, rather than structural and functional organization of task sets *per se*. While these paradigms study in some detail how task sets persist and change over time, they pay less attention to their microstructure, that is, the way in which the event representations involved in a given task set interact with each other and become shaped by the demands of the task at hand.

In this paper I argue for extending this approach. My aim is twofold. The first aim is to outline a framework for task set representation that addresses both, mapping relations between and content profiles within representations. Essentially, the framework claims that the implementation of a given task set does not only act to establish appropriate mappings (*between* representations of pertinent events like stimuli, responding actions and action effects), but also appropriate feature profiles (*within* event representations). In fact, it even argues that mappings between representations are instantiated through overlap of their feature profiles. The framework is derived from Ideomotor Theory and the Theory of Event Coding (TEC). Both view stimuli, responses and response outcomes as commensurate events that share a common representational domain (Prinz, 1987, 1990, 1997; Hommel et al., 2001; Prinz et al., 2009; Memelink and Hommel, 2013).

The second aim is to apply this framework to scenarios in which the task is embedded in a minimal social context. To pursue this aim, we will study experimental settings in which a reaction time task is shared between two participants (namely, *task sharing* settings). Findings from a number of recent studies have been understood to suggest that in such settings participants do not only represent their own share of the task, but also *co-represent* their partner’s share—even if that share is entirely irrelevant for their own performance (Sebanz et al., 2003, 2005, 2006). For instance, when a Simon task is divided between two participants (so that each takes care of one of the two relevant stimulus features and one of the two response key assigned to it), a spatial compatibility effect is observed for each of them—as in the standard version of the task in which a single participant takes care of both stimulus features and their assigned response keys. In contrast, no such effect is obtained when participants perform their task share alone, that is, without another being present. It has therefore been suggested that sharing a task with another invites implementing the full task; representing one’s own share and co-representing one’s partner’s share (Sebanz et al., 2005; Atmaca et al., 2008; Sebanz and Knoblich, 2009). This is where the second aim comes in; to find out what the role of co-representation can be in a broader framework for task representation.

The following discussion will take two major steps. The first step will outline a general framework for task representation, focusing on ways in which task demands shape representational content. The second step will then apply the logic of the framework to various kinds of interference paradigms, thereby gradually moving from unshared to shared settings. As we will see, the notion of co-representation will be lost in doing so, suggesting the radical conclusion that we don’t need it at all (Dolk et al., 2011, 2013; Dolk and Prinz, in press; Wenke et al., 2011).

## A Framework for Task Representation

What does it take to carry out a reaction time task? Essentially, we may discern two levels of task description, external/observational and internal/theoretical. Adopting an external point of view, we may observe certain kinds of events taking place in the experimental setting. Typically, we observe an individual generating certain actions in response to certain stimuli presented to them, thereby following certain rules to which they have committed themselves. For instance, in the Simon task the individual may map red vs. green stimulus patches appearing on the screen to pressing left vs. right response keys mounted on the table, based on rules provided by preceding instructions.

Conversely, adopting an internal point of view, we may address the putative cognitive architecture whose structural and functional features are meant to account for observed performance. Typically, we invoke an architecture harboring stimulus representations, response representations, and linkages established between them. Stimulus representations are understood as internal placeholders for external stimulus events, generated and maintained on the sensory input side of the architecture. Response representations are understood as internal placeholders for external response events, generated and maintained on the motor output side. Finally, linkages between stimulus and response representations are understood as internal placeholders for the stimulus-to-response mappings required by the instruction.

Even though the internal description does not provide much more than an internalized version of the external account, it has over the past decades made its career as a fairly successful theoretical framework for the analysis of cognitive task performance. Building on early pioneers, for example, Broadbent, Posner, Sanders, and Welford, it has meanwhile reached the status of classical textbook wisdom (e.g., Broadbent, 1958; Welford, 1968, 1980; Sternberg, 1969; Posner, 1986; Sanders, 1998). Nonetheless, it has not gone unchallenged. The critical challenge comes from observations on so-called *privileged linkages between perception and action* and related theoretical claims for a functional role of *similarity* in the operations subserving these linkages (Prinz, 1990, 1997; Prinz and Hommel, 2002). The crucial observation here is that stimulus events can often more easily be linked to response events resembling them, as compared to non-resembling ones. For instance, in the Simon task a stimulus patch on the left side of the screen can be more easily and more efficiently linked to a key press response on the left side, as compared to the right side. The same holds true not only for a number of further instances of stimulus-response compatibility effects (e.g., Fitts and Seeger, 1953; Fitts and Deininger, 1954; Alluisi and Warm, 1990; Kornblum et al., 1990; Reeve and Proctor, 1990) but also for instances of action induction and action imitation (Lotze, 1852; Carpenter, 1874; Piaget, 1945/2013; Liberman et al., 1967; Meltzoff and Moore, 1977; Knuf et al., 2001; Prinz et al., 2005; for overviews cf., e.g., Prinz, 1987, 1990; Meltzoff and Prinz, 2002; Hurley and Chater, 2005a,b).

The classical framework has no obvious way to account for such similarity-based linkages between perception and action. This is because it regards linkages between input and output as arbitrary connections between incommensurate representations—incommensurate in the sense that they do not share any features on which the operation of similarity can be established. This is where the TEC and the notion of Common Coding comes into place (Prinz, 1990, 1997; Hommel et al., 2001). They suggest extending the classical framework in a way that, atop of arbitrary linkages between incommensurate representations, also permits similarity-based linkages between commensurate representations.

Common Coding invokes an architecture of two parallel systems mediating between perception and action: a primary system for linking incommensurate sensory and motor representations, and a secondary one for linking commensurate cognitive representations of input events (stimuli) and output events (responses). While the primary system relies on arbitrary, contiguity-based linkages between input- and output- representations, the secondary system also allows for non-arbitrary, similarity-based linkages (*mapping* vs. *matching* between input and output representations).

With these extensions in mind, let us now see how we can apply basic notions of Event Code Theory to issues of task representation and task set formation. In this section we discuss some basic claims concerning structure and function of the required representational machinery. The first, perhaps most important claim addresses the functional locus of that machinery. We posit that flexible cognitive task performance is mainly, if not entirely, organized by the secondary system. Unlike the primary sensory-motor system whose representational structure relies on long-term sensory and motor experience, the secondary, cognitive system is flexible enough to allow for creating short-lived and task-specific representational structures *(task sets)* and, at the same time, penetrable and transparent enough to allow for these structures being shaped and modulated through verbal communication *(instructions)*. Accordingly, our discussion will focus on ways in which the secondary system subserves task representation and performance.

### Event Codes

The Theory of Event Codes (TEC) offers a framework for characterizing the basic structure of event representation in the secondary system (Prinz, 1990; Hommel et al., 2001). TEC views task sets as assemblies of event codes that are organized in specific ways. Thus, in order to understand how these assemblies are organized and how they work, we first need to understand what event codes are and how they interact with each other.

*Representational function—*The basic idea is simple (and perhaps trivial). Event codes are cognitive representations of a variety of things and events that an individual’s mental activity may address. Importantly, event codes may be placeholders for things and events in different modalities of existence (and even non-existence): things that *happen*, or *ought to happen*, in the past, present and future (and even those that *do* *not* *happen*).

For the present discussion we take the workings of event codes for granted, without addressing the intricate issue as to how they may be generated and individuated from the continuous flow of information to which individuals are exposed. While this issue may be difficult to solve for natural scenarios, we may set it aside when discussing experimental settings. Such settings are actually designed in terms of well-defined things and events, so that we may assume that participants generate task-specific assemblies of event codes whose structure mirrors the structure of events that actually happen in these settings. While some of these events are explicitly mentioned in the instruction, others are introduced in other ways that give rise to the formation of pertinent codes. Accordingly, while we do not offer a detailed account of how these codes are generated, we do maintain that assemblies of task-specific event codes reflect the basic structure of task-specific events.

To fulfill their representational function, event codes need to be equal and mutually commensurate, even if the things and events they represent are incommensurate. How can this be possible? For instance, how can such diverse things like external stimulus events that are controlled and generated by the experimenter (e.g., a color patch appearing on the screen) and internal response events that are controlled and generated by the participant (e.g., her hand pressing a response key) be represented in a common format? As has been argued elsewhere (Prinz, 1992; Hommel et al., 2001; Memelink and Hommel, 2005, 2006, 2013; Prinz et al., 2009), *distal reference* is the key feature here. Event codes are commensurate in virtue of referring to distal events in the world and body. They are, in other words, placeholders for external events outside the representing system, but not for internal events that instantiate their representational function in that system (be it at the proximal level of sensors/effectors or the central level of the brain).

*Structure—*A convenient way of conceiving the structure of event codes is to regard them as *feature compounds* in semantic space (Hommel, 1997, 2009, 2013; Hommel et al., 2001). According to this view, event codes are instantiated in local, transient feature networks that are generated from resources provided by a global, permanent space of semantic features. For instance, a color patch on a screen may be represented by a compound of features, for example, *red, round, small, at-top-of-screen, on-left-hand-side*, etc. The formation of event codes thus requires and presupposes the existence of a global space of semantic features that provides the building blocks for their making. Notably, this space is semantic in the weak sense of codes being grounded in sensory and motor activity, not in the strong sense of codes serving as symbolic placeholders for external events.

**Figure 1:**
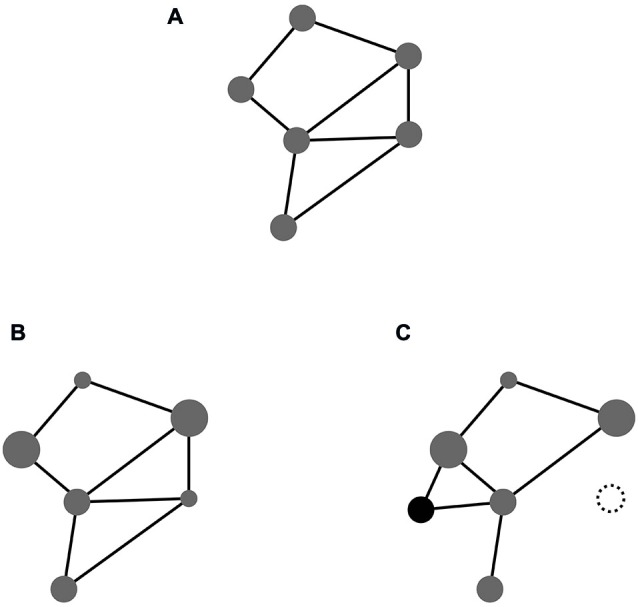
**(A)** Feature compound with equal weights; **(B)** Same compound with different weights (indicated by node size); **(C)** Same compound with one of the original features deleted (dotted) and a novel feature included (black).

Taking these building blocks for granted, we might examine how event codes are formed from them. To this end we outline their elementary structure and discuss ways in which they may change over time. A basic outline is depicted in Figure 1A. It shows a hypothetical feature compound which is characterized (i) through a particular set of features involved in it; and (ii) a particular pattern of connectivity interlinking them. It goes without saying that this sketch must be understood as an abstract, highly impoverished illustration of the underlying idea. The complexity of true event codes will exceed that of the sketch by several orders of magnitude (in terms of both, number of features and complexity of connectivity).

At this point it should not go unnoticed what the sketch fails to show. It fails to show that the feature compounds instantiating event codes are local structures embedded in a global space. This embeddedness has important implications. In the same way as any given code comprises a number of features, any given feature contributes to a number of codes. As a result event codes may overlap with respect to their features. As we will discuss below, such feature overlap plays a crucial role for interactions between event codes in task set formation.

A structure such as the compound depicted in Figure 1A can undergo various kinds of changes (Memelink and Hommel, 2013). While some such changes may pertain to the pattern of connectivity among given features, others, on which we focus here, pertain to the profile of their contributions to the compound. So far we have described in inclusion of features in event codes as an all-or-nothing affair. Figure 1B shows a somewhat more realistic picture, assuming that features may differ in their relative contributions to a given code. While some features may be defining and essential, others may be more or less an accessory for representating an event. For instance, the color of the patch on the screen may be crucial to the task at hand whereas its shape may be irrelevant. Accordingly, when characteristics of things and events become relevant or irrelevant in particular settings, this will alter the relative weights with which corresponding features contribute to the event codes representing them. The pattern of feature weights thus specifies what we may call the *weight* *profile* of an event code. Below, we discuss ways in which that profile may become modulated by task context.

Restructuring weight profiles may be regarded as a weak way of altering the content of feature compounds. A more effective way is to alter *feature profiles* themselves, that is, to include new and/or delete old features altogether, as illustrated in Figure 1C. In one sense these alterations may also be considered weight changes (viz., down to zero and up to a value above zero, respectively). Yet, in another sense they have deeper implications than just weight alterations. This is because they furnish feature compounds with an enormous degree of structural flexibility; the capacity to grow and shrink on demand. As we will see below, this capacity turns out to be of utmost importance for tailoring the content of event codes to the demands of the task at hand.

*Interactions—*If feature weights and profiles can be modulated that way—what factors are modulating them? The basic claim here is that the structure of code profiles at a given time is mainly (if not entirely) determined by the history of interactions between these codes. According to this view, any event code that is involved in the representation of a given scenario becomes selectively tuned to the needs of efficient interaction with other codes involved in the same scenario.

**Figure 2:**
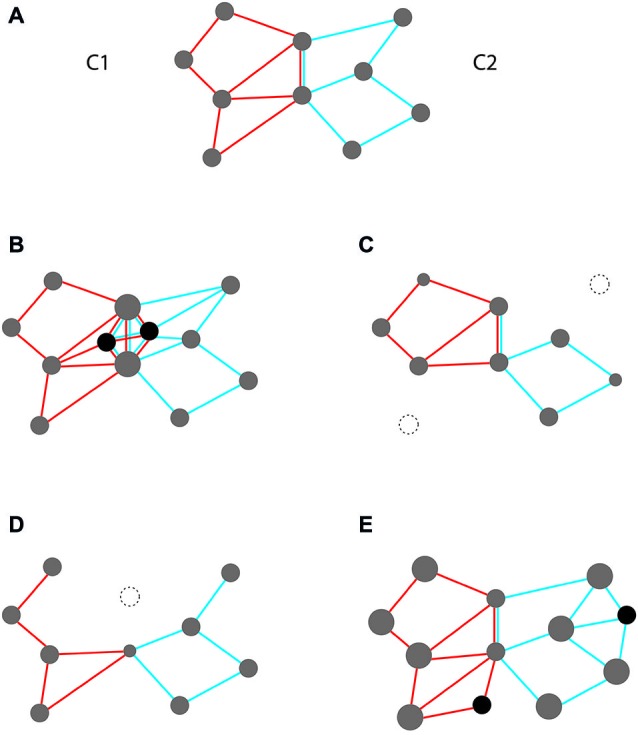
**Two overlapping event codes, instantiated as intersecting feature compounds (C1 and C2 with red and blue edges, respectively)**. Overlap is indicated by feature nodes on which red and blue edges converge (= shared features). **(A)** Initial scheme with equal feature weights. **(B–E)** Possible structural changes under conditions of code cooperation **(B,C)** and code competition **(D,E)**. **(B)** Strengthening shared features through (i) increasing weights of old features (node size); and (ii) including new features that are shared (black); **(C)** Weakening unshared features through weight reduction and deletion (dotted); **(D)** Weakening shared features through weight reduction and deletion; **(E)** Strengthening unshared features through weight increase and inclusion of novel features.

Consider for illustration a simplified scheme with two intersecting and interacting event codes (Figure 2A). Each code exhibits both shared and unshared features. While shared features are common to both codes (and, hence, non-distinctive), unshared features are specific for each of them (and, hence, distinctive). Depending on the interaction in which the two codes are involved, such overlap may either be beneficial or detrimental. Overlap should be beneficial in scenarios requiring code cooperation, i.e., that both are jointly activated at the same time. In contrast, overlap should be detrimental in scenarios requiring code competition, i.e., that only one of them is activated (and the other silenced) at the same time. While cooperation requires that activating one code partially co-activates the other, competition requires that one must be shielded from being co-activated by the other.

The scheme depicted in Figure 2A invites obvious measures for improving the efficiency of both cooperation and competition. These measures rely on altering the structure of code profiles according to given requirements. Measures supporting cooperation are shown in Figures 2B,C. They may pertain to the overlap zone (strengthening weights and creating new features that are shared) or to non-overlap zones (weakening weights and deleting unshared features). Likewise, measures supporting competition are shown in Figures 2D,E. Again, they may pertain to the overlap zone (weakening weights and deleting shared features) or to non-overlap zones (strengthening weights and creating novel, unshared features).

In sum, we see two basic factors at work in tailoring profiles of feature assemblies to context conditions: initial pattern of overlap (shared vs. unshared features) and required mode of interaction (cooperation vs. competition). We claim that these two factors enter learning algorithms for optimizing the efficiency of event codes for processing under given conditions of overlap and interaction. As we will see in the subsequent section, these algorithms play an important role as tools for the formation of task sets. To understand this role we do not need to specify how they work in detail. The only thing we need to accept is that they exist and perform their function.

### Task Sets

The application of these ideas to task sets and task set formation is fairly straightforward. As indicated above, the concept of task set refers to the representational underpinnings of performance in S-R mapping tasks. Our framework claims that event codes provide the basic equipment from which task sets are made. More specifically, it regards task sets as particular kinds of assemblies of such codes. Of these assemblies we demand that they must be organized in ways that allow us to derive basic patterns of task performance from basic patterns of code interaction.

The classical approach to capturing the functional logic of S-R mapping tasks addresses three fundamental sets of task components (cf., e.g., Garner, 1962, 1974; Kornblum et al., 1990; Kornblum, 1992): *stimulus set* (the set of possible stimulus events), *response set* (the set of possible responding actions), and *mapping rules* (the set of prescriptions for assigning responding actions to stimulus events).

While these components address task structure and off-line implementation, basic operations address task performance and on-line execution: *stimulus* *presentation* (presenting an element from the stimulus set) and *response selection* (selecting an element from the response set). Seen from the participant’s perspective, stimulus presentation is under external control (i.e., the experimenter’s protocol), whereas response selection is under internal control (i.e., the participant’s action decision).

**Figure 3:**
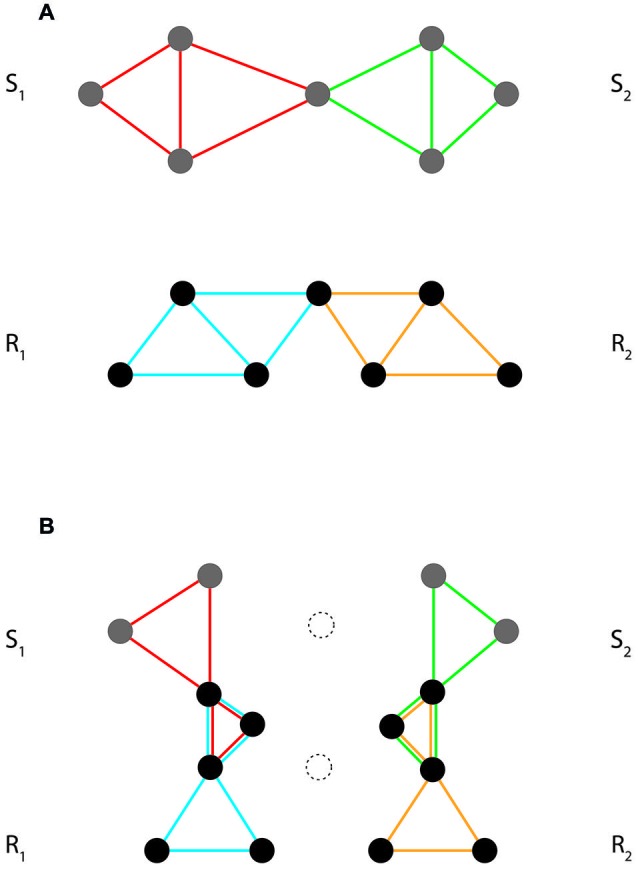
**Interactions between event codes in task set formation. (A)** Initial structure of a hypothetical event code assembly, with two partially overlapping stimulus codes (S_1_/S_2_) and two partially overlapping response codes (R_1_/R_2_). **(B)** Final structure of the same assembly, with initial horizontal overlap *among* stimulus and response codes deleted (unconnected dotted features) and new vertical overlap *between* stimulus and response codes created. Note that the final structure of the assembly differentiates between wanted and unwanted S-R assignments (wanted: S_1_–R_1_ and S_2_–R_2_; unwanted: S_1_–R_2_ and S_2_–R_1_). To illustrate, consider a task requiring left key presses in response to squares (S_1_→R_1_) and right key presses in response to circles (S_2_→R_2_). An efficient task set must, on the one hand, weaken, or delete features that are shared among the competing stimuli and the competing responses (like, e.g., the *closedness* of the two shapes and the *downward-directed push* of the two key presses, respectively). On the other hand, it must implement the required mappings by creating overlap between distinctive features of stimuli and responses (such as, e.g., *straight lines/left key* vs. *curved lines/right key*) and creating new functional features instantiating these mappings.

Task implementation and performance are thus both grounded in interactions among event codes that are involved in task sets (summarized under the notion of *intentional weighting* by Memelink and Hommel, 2013). As illustrated in Figure 3, these interactions transform the initial structure of event code assemblies into a final, task-specific structure. In essence, we may discern two basic kinds of such interactions, horizontal and vertical. Both aim at optimizing task set structure and function. At the level of implementation they tailor the content profiles of stimulus and response codes to the demands of the task at hand. At the level of performance they strengthen cooperation and weaken competition.

#### Horizontal Interactions

Primarily, a task requires identification of stimulus events and selection of appropriate actions for response. Thus, when viewed in terms of event codes and event code assemblies, each of these two operations seems to entail the selection of one element from a set of competing elements. In terms of the scheme depicted in Figure 3, such selective competition requires horizontal interactions *within* stimulus and response codes, respectively. Stimulus identification selects one particular stimulus code from the set of alternative stimulus codes involved in the task. Likewise, response selection selects one particular response code from the set of alternative response codes.

As discussed above, code competition requires structuring the underlying event code assemblies accordingly. An obvious way of achieving this is to strengthen distinctiveness among competing codes through weakening their mutual overlap. As a result of such distinctive tuning, conflict will be reduced and the efficiency of selection will increase. Importantly, this applies to operations, stimulus identification, and response selection (triggered by external presentation and internal decision, respectively).

#### Vertical Interactions

So far we have not yet addressed the role of mapping rules. Mapping rules are prescriptive. Essentially, they require that activation of particular stimulus codes must be followed by activation of particular response codes. Thus, in order to instatiate these rules, the involved event code assemblies need to establish some kind of selective cooperation between stimulus and response codes. Selective cooperation tunes stimulus codes to assigned actions and/or response codes to assigned stimuli. With reference to the scheme in Figure 3, selective cooperation requires vertical interactions *between* stimulus and response codes. These interactions may take two complementary forms, aiming at facilitating wanted and impeding unwanted mappings, respectively.

When a task comprises *n* stimuli and *m* responses, the total number of possible S-R–mappings equals *n* × *m*. Within the total set of possible mappings, instructions introduce a distinction between two subsets: that of wanted, correct mappings and that of unwanted, incorrect mappings. Whilst wanted mappings yield correct responses, unwanted mappings yield errors. Efficient task sets thus require both, to facilitate wanted und impede unwanted mappings.

*Wanted mappings—*Implementation of wanted mappings requires selective cooperation between particular stimulus and response codes. An obvious way of achieving this is to create selective overlap between the involved codes. As an example, we may once again consider a simple choice task that requires pressing a left vs. right key in response to a red vs. green color patch. A mapping such as this is initially entirely arbitrary. Before the task is administered, linkages between features such as *left–red* or *right–green* do not exist. Pertinent event codes are, at this time, entirely disjunct. This is where instructions intervene. Instructions act to create patterns of selective overlap. These zones of overlap instantiate the required mappings between stimulus events and responding actions. Basically, we may imagine two ways of creating such overlap: integration of old features and generation of new ones.

The first option is to integrate given stimulus features into response codes and/or given response features into stimulus codes. As a result, *red* becomes integrated into the response code for *left* key presses, and/or *left* becomes integrated into the stimulus code for *red* patches. The second option is to generate new features and assign them to stimulus and response codes that are required to cooperate. As a result, the new feature *xyz* becomes integrated into both the stimulus code for *red* patches and the response code for *left* key presses.

At first glance such feature assignments appear to be fairly arbitrary and strange. Yet, we need to remind ourselves that features are freefloaters and not naturally and intrinsically tied to the stimulus or the response domain. Each feature can therefore, at least in principle, become integrated into each kind of code. Furthermore we also need to remind ourselves that features are semantic elements that may not only address physical properties of things and events (such as colors of patches or locations of response keys), but also functional properties (such as locations *belonging to* given colors or colors *belonging to* given locations etc.). Such functional features can be seen to represent the fact that the two events are assigned to each other in the current task.

Instructions thus act to create new overlap, either based on old or new features. In virtue of such overlap, codes for *red* patches will co-activate codes for *left* key presses and likewise will codes for *left* key presses co-activate codes for *red* patches. Of course, this presupposes that the weights of the features that make up for the new overlap are strong enough to warrant such co-activation.

*Unwanted Mappings—*Nonetheless, creating new overlap is just one side of the coin. The other side pertains to deleting old overlap. While selective generation and strengthening of new overlap aims at facilitating wanted mappings, selective deletion and weakening of initially given, old overlap aims at impeding unwanted mappings.

Selective deletion comes into effect under conditions of unwanted shared features. Under these conditions feature overlap arises between stimulus and response codes that are *not* assigned to each other by instructions, so that their co-activation may drive wrong, unwanted responses. A typical example is provided by choice tasks with incompatible assignments between stimulus and response events. Consider a task in which tones presented to the *left vs. right ear* require *right vs. left key* *presses* as responses. In a setting such as this the two tones will initially share strong spatial features with the two keys: left and right tones will invite, as it were, left and right responses. However, since task instructions require the reverse assignments, these natural, compatible mappings (*left-left* and *right-right*) are in conflict with the required incompatible mappings (*left-right* and *right-left*). Thus, in order to implement an efficient task set, two complementary measures must be taken. Initial, natural overlap needs to be weakened or deleted, and new overlap needs to be generated and strengthened.

In any case, incompatible assignments must be expected to yield poorer performance than compatible assignments. This may rely entirely upon initial overlap, without the need to weaken or strengthen anything. This is in fact what numerous studies have shown. Relative to neutral assignments that exhibit no initial overlap, incompatible assignments yield slower responses and more errors, whereas compatible assignments lead to faster responses and fewer errors (Alluisi and Warm, 1990; Hommel et al., 2001). Importantly, this is not only true for overlap of spatial and physical features, but also of semantic and symbolic features (Stroop, 1935; LeMay and Simon, 1969; Hedge and Marsh, 1975; Simon et al., 1981; Virzi and Egeth, 1985; Prinz, 1990; MacLeod, 1991; Hommel, 1997; van Maanen et al., 2009). We may, therefore, conclude that our framework is in line with these basic findings. It accounts for both the detrimental effects of incompatibility and the beneficial effects of compatibility.

### Conclusion

Event code theory views task sets as event code assemblies in which event codes from two different sources interact and shape each other: stimulus codes, generated from external sources and response codes generated from internal sources. In each trial the task requires identification of the given stimulus and selection of the required response. On the one hand, stimulus codes and response codes compete with each other *within* their respective sets, to the effect of weakening and deleting initial code overlap (*horizontal interactions*). At the same time particular stimuli have to be mapped onto particular responses, which requires cooperation *between* stimulus and response codes. Selective strengthening of vertical overlap facilitates wanted assignments, whereas selective weakening impedes unwanted assignments (*vertical interactions*).

Importantly, these interactions apply to two time scales: on-line performance and off-line implementation. While performance-related operations apply to *performing the task* (online execution: identification, mapping, selection), implementation-related operations apply to *setting the task* (offline learning: shaping code profiles according to task demands).

One way of viewing this framework is in terms of attentional mechanisms. Similar mechanisms have been proposed to account for selective attention. For instance, the idea of code competition and selection is widespread in the attentional literature (e.g., Logan, 1988, 2002, 2007; Bundesen, 1990, 1998; Desimone and Duncan, 1995; Schneider, 2013; Rosenbaum, 2014). The same applies to the notion of codes as feature compounds whose structure and composition may change on demand (Barsalou, 1999; Hommel et al., 2001; Memelink and Hommel, 2005, 2006; Hommel, 2010). Yet, while the basic logic is very much the same, the functional domain is different. Theories of attention apply these ideas to the domain of perceptual processing, but our framework applies them to the broader domain of interactions between perception and action in task representation. Thus, when viewed from an attentional perspective, our framework can be seen to deal with attentional mechanisms operating in task implementation and performance.

## Application to Interference Paradigms

In this section we move on to apply the framework of event code theory to more complex tasks where the number of stimuli exceeds the number of responses mapped to them. In a task scenario such as this the elements of the stimulus set may differ in features that are irrelevant for response selection. For instance, when stimuli are color patches that can either be red or green and, at the same time, either take the shape of a circle or square, the resulting set of possible stimulus events comprises four elements that differ on two feature dimensions, namely color and shape. According to instructions, color may, for instance, be relevant for response selection (i.e., determine the choice between left and right key presses), whereas shape may be irrelevant (or *vice versa* under reverse instructions).

For a task such as this, our framework leads us to expect (among other things) that relevant features become strengthened in the underlying stimulus codes, whereas irrelevant features become weakened, or even deleted. As a result, response selection should become mainly, or even exclusively, controlled by relevant features. Yet on the other hand, if it appears that irrelevant features cannot be entirely silenced or deleted, their unwanted processing may also impact response selection.

### The Logic of Interference

Interference paradigms are designed to provoke such impact. In typical interference tasks stimulus and response codes overlap with respect to features that take two conflicting roles at a time: irrelevant elements in stimulus codes (requiring weakening) and relevant elements in response codes (requiring strengthening). An example is provided by the Simon task (Simon and Rudell, 1967; Simon, 1968, 1969, 1990; Umiltà and Nicoletti, 1990). In this task participants are required to select one of two keys, for instance in response to the color of a stimulus patch which may appear on the *left-* *or* *the* *right-hand side* of the screen. Color is thus a relevant feature of stimulus events, wheras their location on the screen is entirely irrelevant in the sense that it should play no role for response selection at all. Yet on the other hand, the location of the key to be pressed (mounted on the *left- or the* *right*-*hand side* of the table) is the crucial distinctive feature in which the two competing actions differ. The task can therefore be seen to instantiate a conflict between two roles of a feature: one and the same feature (e.g., being located *on the right-hand* *side*) is entirely irrelevant for stimulus identification, but highly relevant, if not indispensable for response competition.

The pronounced interference effect that is regularly observed in this task indicates that participants find it in fact impossible to effectively ignore the location of the color patch. Instead, the effect thus suggests that the required weakening/deletion of the irrevant stimulus feature does not occur. It looks as if the strong role that this feature plays for response selection spills over into stimulus identification.

**Figure 4:**
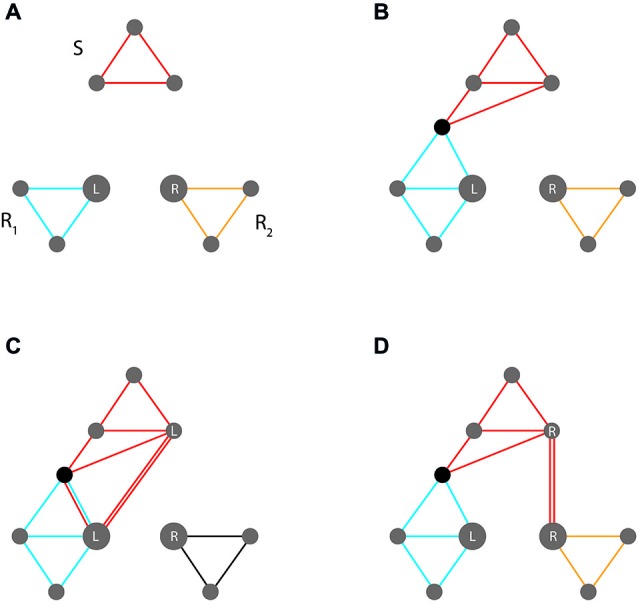
**Event code assemblies involved in the Simon task. (A/B)** Control condition (where stimulus location is constant across trials) **(A)** Initial assembly of non-intersecting stimulus and response codes (stimulus code S and two competing response codes, R_1_ and R_2_, with location features L/R highlighted). **(B)** New overlap between S and R_1_ instantiates the required mapping. **(C,D)** Interference condition (where stimulus location varies across trials) **(C)** Compatible trial; stimulus location (L) supports the required response. **(D)** Incompatible trial; stimulus location (R) supports the opposite response. See text for further explanation.

This dilemma is illustrated in Figure 4. The upper panels show a stimulus code and two competing response codes (e.g., representing a red patch and a left vs. right key press, respectively). Location features in response codes are highlighted, reflecting their importance for response competition. Panel (b) indicates the wanted assignment, indicated by a novel feature that is shared by the stimulus code and the assigned response codes (e.g., instantiating the prescription to press the left key in response to a red patch).

While the upper panels address trials in which the stimulus is presented in a neutral location, the lower two address compatible vs. incompatible trials in which stimuli occur in locations that do or do not correspond to response locations, respectively. Since location features exhibit high weights (arising from response competition), they become automatically (and unavoidably) integrated into pertinent stimulus codes. This will either strengthen the activation of the wanted response or induce activation of the competing unwanted response (in the compatible and the incompatible case, respectively). Relative to neutral trials, response conflict is thus reduced on compatible trials and increased on incompatible trials.

Accordingly, the crucial factor driving the Simon effect derives from a dilemma inherent in the requirement of forming efficient task sets. Efficient response competition requires strong weights for features in which competing responses differ. At the same time efficient stimulus identification requires weak weights for features that are irrelevant.

As long as the features to which these conflicting requirements pertain are different (e.g., stimulus *shapes* and response *locations*), no conflict will arise. However, conflict will become unavoidable, when they are the same (e.g., stimulus *locations* and response *locations*). Stimulus- and response codes will then overlap with respect to theses features and the requirement of strengthening their weights for one code will counteract that of weakening their weights for the other code. In particular, a strong role that a feature plays for the formation of efficient response codes will automatically strengthen its role for stimulus code formation, thus preventing it from becoming weakened or deleted, as optimal task set efficiency would require. As a result, stimulus identification will interfere with response selection.

To examine the explanatory power of this simple principle we now move on to study a variety of interference tasks. The tasks that are included in our examination differ in two major respects: *mode of response competition* and *type of overlapping features*. Event code theory posits that these two factors must act as modulators of task set formation and ensuing performance in interference paradigms.

A special focus of our examination will consider social paradigms—that is, paradigms in which a task is shared between two participants. As will become apparent below, these paradigms are certainly special in the sense of relying on special modes of response competition and special types of interfering features. Nonetheless, our framework can account for them without introducing any special assumptions. (cf. Dolk and Prinz, in press; Dolk et al., 2011, 2014a).

### Modes of Response Competition

Our framework considers response conflict a crucial driving force of task set formation and the ensuing competition a critical constraint of task set structure. Accordingly, we must expect that the mode of that competition impacts both; task set formation and task performance. Here we examine three major types of response conflict and competition: choice, selective response, and joint selective response.

Choice tasks require decisions between alternative actions. For instance, in the case of binary choices on which we concentrate here, choice tasks require decisions between two actions that are both explicitly represented. Since one of them has to be chosen and performed on each given trial, we address them as *Go/Go tasks*. In contrast, selective response tasks require decisions between performing and witholding a single, explicitly represented action. Since one of these two options has to be chosen on each given trial, we address them as *Go/No-go tasks*. Finally, joint selective response tasks combine two individuals performing two complementary selective reponse tasks. In these tasks, too, both individuals have to make choices between responding vs. withholding. However, since their choices are complementary, the combined task amounts to a choice between individuals, that is, *which* of them responds on a given trial. Therefore, we may address such tasks as *I-go/You-go tasks*.

While choice and selective response tasks are classical tools of mental chronometry (Donders, 1868/1969; Wirth, 1927), the joint selective response task is a newcomer to the field (Sebanz et al., 2003, 2005, 2006). In the following sections we concentrate on applications of these tasks to interference paradigms, comparing them in terms of requirements for response competition and ensuing implications for task implementation and performance. In this discussion we will mainly focus on the Simon task as a prototypical interference paradigm and a testcase for examination and comparison.

#### Choices: *Go/Go* Tasks

Classical interference tasks are choice tasks, instantiating competition among a set of responses mapped to a set of stimuli. Here we may confine ourselves to summarizing previously discussed topics from ealier sections. As we have seen, a typical Simon task requires selecting and pushing one of two keys in response to the color, for example, of a stimulus patch on the screen. Interference may then arise from overlap between features of required responses (e.g., *left* or *right* key presses) and irrelevant features of presented stimuli (e.g., *left* or *right* on screen). Since the overlap supports correct/wanted responses on compatible trials, but incorrect/unwanted responses on incompatible trials, it facilitates and impedes response selection accordingly. The resulting performance difference produces the Simon effect.

Choice interference tasks thus instantiate the above-mentioned dilemma between the efficiency requirements of stimulus and response code formation. Features that must play a strong role in one kind of code cannot be weak or even absent in the other. Given the structure of choice tasks, there is no obvious way to resolve the dilemma. This may in fact account for the remarkable persistence of the interference effect in such tasks (MacLeod and Dunbar, 1988; MacLeod, 1991; van Maanen et al., 2009).

#### Selective Responses: *Go/No-go* Tasks

Let us now see what our framework predicts when we alter the requirements of response competition. Consider a *Go/No-go* version of the Simon task in which participants again respond to one of the two colors (*say, red*), but withhold responding to the other color (*say, green*). In a setting like this the stimulus set comprises the same four combinations of (task-relevant) colors and (task-irrelevant) locations as before. In contrast, the structure of the response set is entirely different. Each trial requires a decision between two options: *to push* or *not push* the response key. Conflict and competition thus apply to the options *Go vs. No-go*, that is responding vs. withholding response. Recent evidence has shown that processing mechanisms for withholding are closely related to those for responding, so that withholding a response can also be regarded as a response (Kühn et al., 2009; Kühn and Brass, 2010). If one adopts this view, the *Go/No-go* task may still be seen to instantiate a conflict between two competing response options. Yet, the two options are not spatially distinct anymore: they do not differ in terms of spatial locations to which they pertain.

Our framework predicts that the Simon effect must disappear under these conditions. Since there is no response competition that highlights location features anymore, there is no strengthening of feature weights and, hence, no mandatory inclusion of location features in pertinent stimulus codes. Instead, these features can now be weakened or deleted, as the instruction to ignore them suggests. This is in fact what severalstudies have shown: the Simon effect is abolished in the *Go/No-go* task (e.g., Hommel, 1996; Ansorge and Wühr, 2004).

At this point we may tentatively conclude that event code theory offers a framework that helps us to understand the functional difference between *Go/Go* and *Go/No-go* versions of the Simon task. More specifically we may conclude that the mode of response competion inherent in these versions drives task set formation and determines task set structure.

#### Joint Selective Responses: *I-go/You-go* Tasks

Can the framework likewise account for performance in the joint selective response task? Before we address this question, let us first see what the task requires and how it works. The literature offers two basic accounts to capture the functional logic of the task: individual and social. They differ not only in descriptive terms, but also suggest different explanations that are associated with fairly diverging theoretical backgrounds (Dolk and Prinz, in press; Wenke et al., 2011; Dolk et al., 2014a).

*Individual account—*According to the individual account, the task combines two participants who are required to perform two independent, complementary *Go/No-go* tasks. Typically, the two are seated next to each other, with one of two response keys assigned to each of them. Instructions may require, for instance, that one responds to green, but withholds to red stimuli whereas the other does the reverse, that is, responds to red, but withholds to green stimuli. Each participant’s share of the task is thus, in terms of task requirements, completely equivalent to the regular, single *Go/No-go* task as discussed above. In other words, for each of them response competition is competing between the options of *pushing* or *not pushing* one and the same response key. Accordingly, the individual perspective leads us to expect that the joint selective response setting should exhibit similar interference effects as the regular, single *Go/No-go* setting.

*Social account—*The social account takes a broader perspective. Essentially, it regards the two individual *Go/No-go* tasks as two complementary components of a common *Go/Go* task that is shared between two individuals. Within this common task each of the two paricipants is responsible for one of the two stimulus colors and one of the two response keys assigned to them. Since stimulus color dictates which of the two response keys must be pushed, the full task is seen to instantiate conflict and competition between two spatially distinct response options. Accordingly, the social perspective leads us to expect that the joint selective response setting should exhibit similar interference effects as the regular, single *Go/Go* setting.

As indicated, the two accounts are rooted in quite divergent theoretical and metatheoretical backgrounds (cf. Prinz, 2012, Ch. 2). In particular, they differ deeply in terms of their implied beliefs regarding the nature and representational basis of human sociality. The individual account believes in solipsistic closure. It regards human agents as closed, encapsulated systems that, when brought into social scenarios, tend to concentrate on their own agenda, without taking much notice of others or even coordinating or sharing their agendas with them. In contrast, the social account believes in open-mindedness. It regards agents as open, interactive systems that, when brought into social scenarios, are keen on taking notice of others and opening their own agenda for coordinating and sharing it with them. Sociality is thus a primary, constitutive ingredient of human nature for the social account, while it plays only a secondary, derived role for the individual account.

*Combined account—*Setting such big issues aside, we now take a more pragmatic look at the joint task from the perspective of event code theory. This framework suggests an account that combines individual and social elements (Baess and Prinz, in prep.; Dolk and Prinz, in press; Wenke et al., 2011; Dolk et al., 2014a).

The combined account once more builds up on response conflict and competition as the key factor driving task set formation and shaping task set structure. It combines two elements, *What* and *Who* (Jeannerod, 1999, 2003, 2006; Decety and Chaminade, 2003). On the one hand, it claims that response competition takes the form of a *What-*conflict: in each trial participants decide between responding and withholding—as the individual account suggests. On the other hand, it claims that the *What-*conflict is superimposed by a *Who-*conflict, that is, a conflict between the two agents—as the social account suggests.

The interindividual conflict becomes particularly apparent when we focus on overt responses. When viewed in terms of overt events, trials can be seen to instantiate choices between the responding agents: either I respond or You respond. For each participant, though, this interindividual conflict is perfectly correlated, and hence completely confounded with the individual *Go/No-go* conflict: when I go You withhold, and when You go I withhold. The task thus conflates two modes of response competition: individual selective response and interindividual choice. This is why we may refer to it as an *I-go/You-go* task.

Under such conditions predictions of task performance are difficult to make. Basically, we must expect that task performance exhibits signatures of both modes of response competition. Mixed evidence along these lines is indeed what studies of the joint selective response task show (cf. Dolk and Prinz, in press).

On the one hand, there is strong evidence in support of the claim that the joint task is, in functional terms, still a selective response task, not a choice task. This conclusion is suggested by comparisons of response speed. As is known since Donders’ pioneering studies, response times for choices are substantially longer than for selective responses. The difference may range in the order of up to 200 ms—in fact a gigantic amount of time in terms of the standards of mental chronometry. Given this background, the observation of joint selective responses being substantially faster than choices and exhibiting the same time regime as regular selective reponses, seems to suggest that the embedding of *Go/No-go* tasks in joint settings does not alter the workings of the underlying task set machinery in a fundamental way. It would appear that joint selective responses are processed in the same way as regular selective responses, unlike choice responses.

On the other hand, evidence also exists in support of the claim that the joint task must be regarded as a choice task. This is because a Simon effect is regularly observed in this task. The size of the effect is weaker than in classical choice tasks, but it is highly reliable (Sebanz et al., 2003, 2005, 2006). We may therefore conclude that interference arises in the joint, but not in the regular version of the selective response task. This seems to indicate that individual task set formation becomes modulated by social context.

Our framework can readily account for these observations and conclusions. The central claim is that social context acts to shape response code assemblies, which then act back on stimulus code assemblies and interactions between the two.

The critical contribution that social context makes in shaping competing responses comes from integrating new distinctive features. In the joint task such new features can be derived from the fact that the two competing responses are distributed between two agents who differ from each other in several respects. Accordingly, any feature in which the agents and/or their responses differ is now a candidate for becoming included in their respective response codes. The joint task thus offers new distinctive features for inclusion in response codes—features that do not exist in the regular individual task.

Location features provide an obvious example. When two agents share a Simon task, they are seated on two chairs next to each other in front of the screen. They are, in other words, distinguished by their spatial locations in the scenario. Relative to the other, one is the *left* and the other the *right* agent. Accordingly, these features offer themselves for inclusion in the competing response codes. If this happens they will, on the one hand, increase code distinctiveness and improve the efficiency of response selection (as illustrated in Figure 2E). However, if they overlap with irrelevant stimulus features, they will also give rise to interference (as illustrated in Figures 4C,D), resulting in a (albeit weak) Simon effect. Here we return to the dilemma inherent in interference tasks: interference is the consequence of the same feature requiring inclusion in response codes and deletion from stimulus codes.

Looking back, we can now specify more precisely the way in which the combined account integrates individual and social elements. There are two basic claims. The first is that individuals concentrate on representing their own share of the task, without representing the other agent’s share. This is the individual element. The second is that they still form representations of agents and actions involved in the task scenario, exploiting them for shaping and optimizing their own task sets. This is the social element (Dolk and Prinz, in press; Dolk et al., 2014a).

In sum, the combined account does not believe in the romantic idea of task co-representation, claiming that agents share common representations of the full task in which they participate. The combined account posits *shaping* rather than *sharing* task sets. This notion reflects the pragmatic idea that representations of co-actors and their actions help agents to shape representations of their own task (Dolk and Prinz, in press).

### Types of Overlapping Features

Up to this point we have treated interference paradigms as a uniform category and discussed the Simon task as a prototypical example. Yet, when we consider a broader range of tasks, we need to extend our framework to account for different types of overlapping features and patterns of interference effects associated with them.

More specifically, we may discern three major paradigms that differ in terms of the stimulus-related features whose overlap with response-related features gives rise to interference: the Simon task (where overlap pertains to spatial locations), the Eriksen flanker task (where it pertains to categorical assignments), and a face perception task (where it pertains to personal identities).

#### Locations

Since we have used the Simon task as a testbed for applying event code theory to interference paradigms, we may start summarizing what has been discussed hitherto concerning spatial interference effects. Interference in the *Choice* version of this task arises from overlap between spatial features associated with stimuli and responses (i.e., *left* vs. *right* locations). This overlap acts to facilitate and impede response selection on in compatible and incompatible trials, respectively. The resulting difference produces the Simon effect.

In the following, we focus on *Selective Response*s. Firstly, we discuss fundamental mechanisms of spatial interference. Here we consider both, *Individual* and *Joint Selective* *Response* tasks. Secondly, we go beyond basic mechanism, focusing on the impact of *Social Modulators* on spatial interference.

*Individual selective responses: Go/No-go—*As we have seen the interference effect disappears in the *Individual* *Selective Response* version of the Simon task. Our framework offers a simple explanation for this observation, based on the fact that in *Go/No-go* task response conflict applies to options that do not differ in terms of spatial locations (viz., *pushing vs. not pushing* a given response key). Thus, since response codes do not carry location features, overlap between stimulus and response locations cannot emerge, and spatial interference effects cannot be obtained.

This view claims that the crucial difference between *Choice* and *Selective Response* lies in different roles of spatial response features. These features play a strong role in *Choice*, but no role at all in *Selective Response*. An obvious way of testing this claim is to run a *Go/No-go* task scenario where spatial layout gives rise to localizing the involved response key and/or the act of pushing it on a *left-right* dimension. This should furnish the underlying response code with corresponding location features.

One way of achieving this is to introduce an accessory reference object or event and integrate it into the spatial layout of the task scenario. If the object or event is sufficiently salient, it should act as a reference for localizing the key press on the left/right dimension, thus furnishing the underlying response code with a location feature pertaining to that dimension. The accessory reference is thus expected to play a similar role for *Selective Responses* as the competing response does for *Choices*: to lay the ground for referential coding of response locations and feature overlap and interference arising from such coding.

This prediction has been confirmed in recent studies. Dolk et al. (2011) showed that a Simon effect in a *Go/No-go* task cannot only be obtained when a co-actor is introduced (i.e., under *Joint Go/No-go* conditions; see below), but also when, instead of their co-actor’s response key, another salient object (e.g., a Japanese waving cat or a ticking metronome) is mounted on the left- or right-hand side of the participant’s response key (Dolk et al., 2013; see also Dolk et al., 2011). These studies demonstrate that any attention-capturing object may serve the function of a spatial reference for furnishing response keys with location features.

These findings support the idea of whether spatial interference is obtained in the Selective Response task and whether it is determined by the spatial layout of the task scenario. If the scenario harbors a salient reference, participants will localize their responses accordingly, thus furnishing their response codes with location features. Once established, location features in response codes will give rise to spatial interference, due to their overlap with location features in stimulus codes.

*Joint selective responses: I-go/You-go—*The scope of this explanatory approach is evidently not restricted to the individual task. It can also be applied to the *Joint Go/No-go* task. In fact it was initially conceived to account for the *Joint* task and only then tested on the *Individual* task (e.g., Guagnano et al., 2010; Dolk et al., 2011).

Essentially, this approach treats actors, co-actors and actions as physical objects and events that are specified by their locations in the spatial layout of the task scenario. More specifically, the claim is that the co-actor and/or his response serve as a reference for localizing the actor and/or her own response—provided that the co-actor’s involvement in the task is sufficiently salient. For instance, it has been shown that interference is obtained when the co-actor is actively involved in the task, not when he/she is passively sitting by the actor’s side (Sebanz et al., 2005, 2006). Likewise, interference seems to depend on distance of the reference: a Simon effect only obtaines when the co-actor is seated close-by, not when he/she is seated at a distance (Guagnano et al., 2010).

Referential coding can thus account for the emergence of interference in the *Joint Go/No-go* task. However we should realize that the notion of referential coding is grossly underspecified. For instance, we are unclear as to which objects and events qualify as references. There are several candidate items, such as response keys and their sounds, arms, hands and fingers as they operate response keys, torsos to which these limbs are attached, and seats carrying them etc.

Under normal conditions the relative locations of these items are perfectly correlated: a response key on the left is operated by a hand on the left, which is, in turn, attached to a torso on the left, etc. Still, we have limited knowledge as to their respective contributions to spatial coding; does location coding mainly pertain to response devices—or to agents and their bodies? First evidence seems to suggest that efficient location coding may in fact involve both, keys and seats at the same time (Dittrich et al., 2013).

*Social modulators—*So far we have concentrated on co-actors and their actions as physical objects and events. Yet, the combined approach outlined above adopts a more comprehensive view that goes beyond the physical domain. As have seen, this approach posits that the *What-*conflict between *Go* and *No-go* is in the *Joint* task superimposed by a *Who-*conflict between *I* and *You*. Accordingly, it predicts a critical role for what we may call *Self/Other overlap*: any features in which the two agents differ or overlap can be expected to play a functional role in resolving the combined *I-go/You-go* conflict.

According to this view, spatial location is just one such distinctive feature. Beyond their accidental seating in the experimental scenario, the two agents will also differ in a number of non-spatial and non-physical respects, be it in terms of transient states or permanent traits. Thus, when two individuals meet to engage in a joint task, they will immediately form impressions about each other, including intuitions concerning *Self/Other overlap*. These intuitions will adress both, features that are common to them (e.g., female, German, tall) and features that distinguish them (e.g., dressed in black vs. white, speaking with Bavarian vs. Saxonian accent, friendly vs. unfriendly behavior, etc.).

Taking this into account, their seating in the experimental scenario does no more than add one particular transient state to that list, viz. sitting *left vs. right* of the other. Even if we assume that such spatial features are coded automatically, their contribution to the formation of pertinent response codes are likely to depend on their relative role in the total list of distinctive features instantiating *Self/Other overlap*. The shorter the list, the stronger their contribution will be, and *vice versa*: When the two agents resemble each other in many respects, the list of distinctive features will be short, so that the added value of distinctive location features will be substantial. Conversely, when the agents differ in many respects, the list will be long, so that the added value of distinctive location features can only be marginal.

Accordingly, the framework predicts that agent similarity, or *Self/Other overlap*, must modulate spatial interference in the *Joint Selective Response* task. This prediction was recently confirmed in several studies. One line of evidence stems from studies combining in-group and out-group members in the *Joint* task. Here it was shown that a Simon effect is obtained when two (mutually similar) in-group members interact, but not when two (dissimilar) outgroup members interact (Müller et al., 2011). A further line of evidence pertains to the role of interpersonal relationships. Factors such as positive mood and positive relationships between co-acting individuals are known to increase perceived self-other overlap (e.g., Davis et al., 1996; cf. Heider, 1958). As we must therefore expect, such factors also act to increase spatial interference in the *Joint* task (Hommel et al., 2009; Kuhbandner et al., 2010).

As a concluding remark it should be noted that social modulation of spatial interference is also predicted by the co-representation account. The claim here is that individuals are more prone and/or more efficient in *sharing common task representations* with similar as compared to dissimilar others. In contrast, our framework relies entirely on *shaping individual task representations* according to the requirements emerging from spatial and social characteristics of the task scenario.

#### Assignments

It is sometimes argued that interference in the Simon task may be special because spatial features may play a privileged role in sensorimotor processing which may perhaps be hardware-rooted. For instance one may argue that spatial interference arises from physical features whose coding requires no more than just discriminating between two well-separated locations of stimulus and response events.

In contrast, semantic interference relies on arbitrary categorical assignments. The Eriksen flanker task (Eriksen and Eriksen, 1974; Eriksen, 1995) provides a paradigm for the study of interference arising from short-lived stimulus-response assignments. In the flanker task participants respond to two (or more) targets (e.g., letters, shapes, etc.) by pressing one of two response keys. Targets are surrounded by distracting flankers that are either (1) the same as the target (identical trials); (2) perceptually different from the target, but assigned to the same response (compatible trials); (3) perceptually different and assigned to the competing opposite response (incompatible trials); or (4) perceptually different and not assigned to any response (neutral trials).

To illustrate, we may refer to a study by Atmaca et al. (2011). In their experiments a string of five letters were presented in each trial: a target in the center, surrounded by four identical flankers (two on either side). In a given trial, one of four letters could appear in the target position: *H* and *K* (assigned to one of two response keys) and *S* and *C* (assigned to the opposite key). As an example for explaining the four task conditions, we may consider trials on which letter *H* served as target. In identical trials the same letter also served for flankers (yielding *HHHHH*), whereas in neutral trials the same function was served by a letter that was never used as target (and therefore never assigned to a response key, e.g., *UUHUU*). These two conditions were considered control conditions. Experimental conditions were provided by compatible and incompatible trials. In compatible trials flankers were physically different from the target but assigned to the same response category (yielding *KKHKK*), wheras in incompatible trials they were both physically and categorically different (yielding, e.g., *SSHSS*).

As with the Simon task this paradigm has generated a large amount of literature pertaining to various task versions and research questions. (cf., e.g., Eriksen, 1995; Purmann et al., 2011; Davelaar, 2013). The common finding is that flanker-related processing interferes with target-related processing, and that it does so despite the fact that task instructions advise ignoring flankers altogether.

Firstly, and perhaps not too surprisingly, response times for target categorization are substantially shorter in identical than in neutral trials, indicating that identical flankers may facilitate target processing. Secondly, and perhaps more interestingly, response times in compatible trials are often faster than in neutral trials, indicating that interference may not only arise from physical features but also from semantic, categorical features. Thirdly, and even more interestingly, responses are usually much slower in incompatible than in neutral trials, supporting and strengthening the same conclusion.

Unfortunately, contrasts involving the so-called neutral condition are less conclusive than this brief summary suggests. This is because two factors are confounded in such contrasts: assignment and valence. The assignment factor refers to the functional contrast of interest here: flankers may be assigned or not assigned to a response. The valence factor refers to a different functional contrast: flankers may be bivalent or univalent. Assigned flankers are always bivalent, that is, they can occur in the task in both roles; flankers and targets. Conversely, unassigned flankers are univalent, that is, they can only occur as flankers throughout the task. Thus, since univalent flankers are certainly easier to ignore than bivalent flankers, neutral trials cannot be expected to deliver a truly neutral reference for compatible and incompatible trials. This is in fact in line with results from most experiments: responses in so-called neutral trials are often as fast as in compatible trials, or even faster.

As with the Simon task we must therefore concentrate on the net compatibility effect, i.e., the contrast between compatible and incompatible trials. This contrast adresses assignments, unconfounded with valences. The sole difference between compatible and incompatible trials pertains to the response category to which the flankers in the string are assigned. In the compatible case both target and flankers drive wanted responses. Conversely, in the incompatible case the target and flankers drive wanted and unwanted responses, respectively. As a result, interference obtains: relative to the compatible case, response conflict is stronger in the incompatible case, weakening performance accordingly.

Flanker interference is grounded on semantic features—semantic in the sense of instantiating arbitrary assignments between stimulus letters and response keys. Notably, these assignments are acquired within the task. It is only by following instructions that participants learn that *H* and *K* belong to one response category and *S* and *C* to the other. In this respect semantic interference is indeed fundamentally different from spatial interference. While spatial interference relies on overlap between physical features that are inherent in the task layout, semantic interference relies on arbitrary stimulus-response assignments that are only established by task instructions. Thus, unlike spatial features that can be derived from overt perception of the task layout, semantic features must be derived from covert knowledge of task-specific assignments.

Our framework offers a simple and convenient way of conceiving the representational underpinnings of that knowledge. The underlying assumption is that new assignments are instantiated through new features. According to this view, implementing new assignments into a task set is tantamount to creating a novel feature that forms part of both, the stimulus code and the response code assigned to it (cf. Figures 2B, 3B). This feature then instantiates the wanted assignment. It instantiates both, the prescriptive assignment that the stimulus must be followed by the response and the descriptive assignment that the response may be linked to the stimulus.

Such features stand for the assignment as such: these are what they represent and what they mean. Their functional significance for the task set will then depend on the relative weights that they attain in the feature compounds that make up stimulus and response codes. The flanker task suggests that verbal instructions can drive their weights to a level strong enough to give rise to substantial interference effects.

*Individual selective responses: Go/No-go—*As seen above, the transition from *Go/Go* to *Go/No-go* is in the Simon task associated with a loss of response-related location features. The loss is critical since spatial interference builds on such features. However, in the flanker task we are facing a different functional situation. In this task semantic assignment features become partially lost and partially preserved on that transition. For instance, when instructions require responding to *H* and *K* and withholding response to any other target, the assignment of *H* and *K* to the response key is preserved, whereas any other letter-key-assignments are lost. Unlike key-related location features, key-related assignments do not require the opposite key as a reference. Thus, when the opposite key is removed, the remaining key looses its location, but not its assignment.

As a result we must expect to see a positive, not a negative compatibility effect in the *Go/No-go* task. There can be no negative effect as there is no longer any functional difference between neutral and incompatible trials: incompatible trials (such as *SSHSS* or *CCKCC*) are, in functional terms, neutral trials. Conversely, the positive effect must be preserved as the covert assignment knowledge on which it relies is unaffected.

In practice, however, the above-mentioned confound forces us to concentrate on the net compatibility effect, that is, the contrast between compatible and incompatible trials. With respect to this contrast we must expect that a compatibility effect should still be obtained. This is because flankers contribute to driving the *Go* response on compatible, but not on incompatible trials. This prediction is confirmed by task performance data (e.g., Atmaca et al., 2011, Experiments 1 and 3).

A further prediction is that the compatibility effect must depend on prior experience with other versions of the task. For instance, when participants perform a *Go/No-go* task *after* performing other task versions in which incompatible flankers were assigned to overt responses, they may be expected to carry over these previously acquired assignments to the new task. An example is provided by Experiment 3 in the study by Atmaca et al. (2011), in which the *Go/No-go* task could be administered either before or after a different version of the task in which the same incompatible flankers were assigned to response keys. While interference was otained in both conditions, it was modulated by previously acquired assignments. The compatibility effect in a current *Go/No-go* task increased substantially when assignments acquired in a preceding task could be carried over to the current task.

In sum, these observations suggest that, unlike the Simon task, some interference is preserved when we move from choices to selective responses. This difference in performance reflects a difference in the features that give rise to interference. While spatial features anchored in the overt task layout are lost in this way, semantic features anchored in covert knowledge are preserved.

*Joint selective response: I-go/You-go—*For this task the combined account claims that the individual conflict between responding and withholding (*Go/No-go*) is superimposed by a social conflict between the two *responding* agents (*I/You*). Agents may differ in many ways. As we have seen, crucial features driving the joint Simon effect pertain to co-actors’ spatial locations. Their overlap with corresponding stimulus features gives rise to spatial interference.

In the flanker task the agents also differ in terms of spatial locations, although in this task there is no overlap between stimulus and response locations. Instead, overlap now applies to flanker assignments. In compatible and incompatible trials flankers are assigned to *I* and *You*, respectively. While the assignment of compatible flankers to oneself also applies to the individual task, the assignment of incompatible flankers to one’s co-actor is only established in the joint task. As a result, flanker interference must be expected to increase in the joint task. As in the individual task, compatible flankers must be expected to help, but atop of this incompatible flankers must also be expected to hurt now.

Experimental evidence confirms this prediction. Net flanker interference is always higher in *Joint Go/No-go* tasks than in *Individual Go/No-go* tasks (Atmaca et al., 2011). Remarkably, this applies to both; tasks involving human and non-human co-actors, suggesting that flankers may not only become assigned to real agents who perform the task, but also to non-performing pseudo-agents (Dolk et al., 2014b). Taken together, the evidence from these studies supports the claim that in the *Joint* task intraindividual conflict between responding and withholding becomes correlated and conflated with interindividual conflict between onself and one’s co-actor.

#### Identities

As a final example we consider a paradigm for the study of interference arising from irrelevant face information (Baess and Prinz, in prep.; see also Philipp and Prinz, 2010). The task required *Go/No-go* decisions, dependent on the color of circles presented on the screen (e.g., *Go* for white and *No-go* for black circles, or vice versa). The circles were superimposed on background faces whose identity were irrelevant for task and therefore had to be ignored. Nonetheless, the critical manipulation pertained to the identity of the irrelevant faces on which the relevant circles were superimposed. Three kinds of trials were randomly intermixed, varying in the identity of the background face and instantiating different degrees of face/agent overlap: *own face, familiar face* and *neutral face*. In *own-face* trials circles were superimposed onto a photo showing the face of the responding agent. In *familiar-face* trials they were superimposed onto photos showing familiar individuals such as co-actors, friends or siblings. *Neutral-face* trials showed entirely unfamiliar faces for control.

*Individual selective responses: Go/No-go—*In this study a reliable *own-face advantage* was obtained throughout: *Go* responses on *own-face* trials were always faster than in *neutral-face* trials. Results for familiar faces depended on the depth of familiarity. When photos showed faces of deeply familiar individuals such as siblings or friends, a *familiarity advantage* was obtained. In this case *Go* responses for familiar faces were just as fast as for own faces. No such advantage was obtained when photos showed faces of superficially familiar individuals such as experimental co-actors. In this case, *Go* responses were just as slow as for neutral faces.

To account for the *own-face advantage*, we need to address the question of what kinds of features faces and Go/No-go responses have in common and what own faces have that neutral, foreign faces don’t have. One kind of such features pertains to *personal identity*. On the stimulus side, the role of identity features is fairly obvious: more than any other body part a person’s face bears the signature of his/her identity. Moreover, since faces are salient pop-out stimuli that are hard to ignore, we may assume that features pertaining to facial identity are automatically processed in this task.

The role of these features is perhaps less obvious on the response side. However, the conflict between responding vs. withholding is conflated with an asymmetry in terms of agent identity. *Go* responses are overt actions that require an agent performing them: when there is an overt action there must also be an agent. Response codes for *Go* responses must therefore specify both, the action (*What*) and the agent performing it (*Who*). Conversely, *No-go* responses do not imply any overt actions that require an agent performing them: when there is no overt action there can be no agent either. Response codes for No*-go* responses can therefore neither specify the action nor the agent.

This asymmetry may explain why *Go* responses are faster on *own-face* trials than in *neutral-face* trials. When the photo shows one’s own face, the identity of the responding agent (as specified by the response code) overlaps with the identity of the stimulus face (as specified by the stimulus code). No such overlap applies when the photo shows a neutral face. This functional difference may give rise to the *own-face* advantage. Its phenomenal counterpart is the intuition in which pictures of participants’ own face entail action impulses that pictures of neutral faces don’t: for them, it is as if seeing their own face prompts their own action.

A related, though more formal type of features that stimulus faces may share with *Go/No-go* responses pertains to *dimensional polarity*. The *Go/No-go* dimension is unipolar in the sense that one response option is well defined (positive polarity: push the key in front of you), whereas the other is defined by default (negative polarity: do nothing). The same applies to the face dimension: one’s own face is singular and well defined, whereas other faces are defined by default. As a result, the positive polarity of one’s own face matches the positive polarity of the *Go* response. Such polarity correspondence may likewise contribute to the *own-face* advantage (Seymour, 1971; Proctor et al., 1992; Proctor and Cho, 2006).

To account for the *familiarity advantage* we may again resort to overlap of identity features. If it is true that response codes for *Go* responses address both, the overt action and oneself as responding agent, we should not be surprised to see that overlap between face and agent identity is not an all-or-none affair, but may be graded according to the social and semantic distance between the face on the screen and oneself as responding agent. Deep familiarity that relies on long-term acquaintance (e.g., siblings or friends) seems to create strong overlap—being no weaker than familiarity with one’s own face. In contrast, superficial familiarity that builds on short-term acquaintance (e.g., experimental co-actors first encountered a few minutes before) seems to be insufficient in creating any overlap at all.

Taken together, the results suggest considering *own-face advantage* as a special case of *familiarity advantage*. In this paradigm interference arises from overlap between identities of stimulus faces and responding agents, and the features instantiating them in stimulus and response codes. Interference in the face task thus relies on features whose assignment to stimulus and response codes builds on previously acquired knowledge. This explains why interference is obtained in the *Individual Go/No-go* task: critical features are derived from internal, knowledge-based resources, not from external, stimulus-based resources (as in the Eriksen task, but unlike the Simon task).

*Joint selective responses: I-go/You-go—*Finally, when we move from the individual to the joint version of the *Go/No-go* task, agent asymmetry (*I* vs. *No-one*) becomes agent competition (*I* vs. *You*). Agent competition should support agent differentiation, that is, increase weights of distinctive features for the identities of the two competing agents. Since these response-related identity features overlap with stimulus-induced identity features, the basic prediction is that identity-based interference should increase when one moves from the individual to joint *Go/No-go* task.

In the study by Baess and Prinz (in prep.) the evidence in support of this prediction was mixed. An increase of *own-face* advantage was in fact obtained when the joint task was shared with a hitherto unknown co-actor. However, no such increase was obtained when the task was shared with a sibling or friend. In other words, the prediction was confirmed under conditions of shallow, but not deep familiarity.

**Figure 5:**
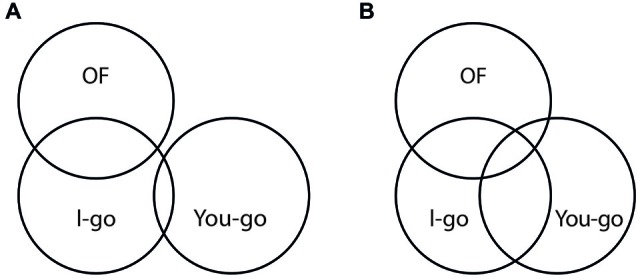
**Interaction between (vertical) face/agent and (horizontal) self/other overlap**. Due to vertical overlap between stimulus and response codes, a photo of one’s own face (*OF*) primes one’s own Go response (*I-go*). As long as horizontal overlap between response codes is weak the competing code (*You-go*) is unaffected by the vertical overlap (panel **A**, unfamiliar co-actors). However, when horizontal overlap is substantial, one’s own face will also prime the competing code (*You-go*; panel **B**, familiar co-actors).

To account for this surprising finding, the authors suggested resorting to the role of familiarity for *Self/Other overlap* (Baess and Prinz, in prep.). When actors and co-actors are closely related to each other (i.e., siblings or friends), their representations of themselves and of their co-actors will strongly overlap. Due to this overlap a photo of their own face will not only prime their own *Go* response, but also their co-actor’s *Go* response to some degree (hence, their own *No-go* response conflated with it). As shown in Figure 5, (horizontal) self/other overlap must thus be expected to modulate the efficiency of priming arising from (vertical) face/agent overlap. Critically, these two factors have opposite effects on response competition. On one hand, response competition decreases as face/agent overlap increases, but on the other hand it increases as self/other overlap increases. The speed of the *Go* response must therefore reflect the combined effect of two counteracting factors that may cancel each other.

At first glance, this finding and the account offered for it seem to be at variance with observations and explanations discussed in the above-mentioned studies on the role of self/other overlap in the joint Simon task. As reported, agent overlap in these studies was always correlated positively with interference. This would lead one to expect strong interference for siblings and friends (where self/other overlap must be fairly strong), but weak or no interference for unknown co-actors (where agent overlap must be much weaker).

A closer look reveals that there is no contradiction at all, however. This is because differences in agent overlap must be associated with differences in the relative contributions of response- vs. agent-related features to the combined response/agent codes. More specifically, an increase in agent overlap must be expected to weaken the weights of agent-related features, to the effect of strengthening the relative weights of response-related features. Such restructuring of feature weights must lead to different consequences for the Simon task and the face/agent interference task. Since Simon interference relies on overlap between stimulus and response-related features, strengthening the role of response-related features must lead to stronger interference. Conversely, since face/agent interference relies on overlap between stimulus and agent-related features, weakening the role of agent-related features must lead to weaker interference. This is why the finding regarding the role of self/other overlap in the face/agent interference task is entirely compatible with seemingly conflicting findings concerning its role in the Simon task.

### Conclusion

Event code theory claims that interference arises in tasks in which the same features adopt two roles at a time: that of irrelevant elements in stimulus codes and that of relevant elements in response codes. In this case conflict becomes unavoidable. Since stimulus and response codes overlap in these features, the requirement of strengthening feature weights for one code must counteract the requirement of weakening them for the other. In particular, must a strong role that features gain from processing irrelevant stimulus information counteract their suppression for efficient response selection? This is the dilemma on which interference builds.

In this section we applied this principle to a variety of interference paradigms, including joint tasks that are shared between two participants. In one aspect, since our framework considers response competition the chief driver of interference, different modes of such competition must give rise to differnt patterns of interference. However, since it also claims a crucial role for features in which stimulus and response codes overlap, different kinds of such features must likewise be associated with different patterns of interference.

Concerning the role of social context for task set formation, two major observations have emerged, one of which pertains to response competition. Task sets for shared tasks seem to instantiate a special mode of response competition in which an intraindividual conflict between responding and withholding is conflated with an interindividual conflict between the two agents, yielding an *I-go/You-go* conflict. The other observation pertains to the nature of features whose overlap gives rise to interference. Such features may, in some paradigms, refer to basic elements for example, key locations, body parts and body movements. However, in other (if not all) social paradigms they also refer to the agents themselves—both physically (e.g., being seated alongside each other) and non-physically (e.g., being related to each other as siblings or aquainted as friends).

Taken together, the two observations suggest a prominent role of agent-related features for task set formation in social context. When a task assigns competing actions to competing agents, task set formation will adress both actions and agents. As a result, response codes will include features refering to the agents who respond, not just the actions through which they respond.

## The Benefits of Matching

As discussed above, the notion of matching plays a key role in the Event Code Theory of task set formation. Unlike mapping operations, which are thought to rely on extrinsic, arbitrary linkages, matching operations are thought to rely on intrinsic, content-based interactions between stimulus and response codes. This raises the issue of how mapping and matching are related.

There are two seemingly contradictory answers to this question, one at the level of representing world events through mind codes, and the other at the level of formation of these codes from elementary features.

The answer at the representational level invokes *mapping-through-matching*. This principle claims that incommensurate objects can become represented by commensurate codes in the mind, so that code similarity, or overlap plays a role in their interaction. In line with this idea, our framework claims that S-R mappings are instantiated through creating selective overlap between stimulus and response codes (as illustrated in Figures 2B,C, 3B, 4B). Once implemented, the overlap mediates interactions between stimulus and response codes in performance. It thus accounts for arbitrary relationships between incommensurate events (e.g., stimulus colors and response keys) through similarity relationships beween their commensurate event codes.

The concept that objects and events that are coupled to each other may be represented by codes whose overlap indicates their coupling, has recently gained support from fMRI studies addressing the shaping of cortical representations through temporal regularities. Results showed that several brain areas encoded regular couplings between objects and events through increasing the overlap of their cortical representations (Schapiro et al., 2012, 2013). These findings suggest a functional role for representational similarity: couplings between objects and events become instantiated through overlap between their representations. Our framework claims that the same principle holds for the coupling of stimuli and responses in task sets.

Mapping-through-matching holds for represention of events in the world through codes in the mind/brain. In contrast, the reverse principle seems to hold for the formation of event codes from features. At this level *matching-through-mapping* applies. Event codes build on local feature compounds that rely on arbitrary mappings of selected features (e.g., their selective inclusion/exclusion or their selective strengthening/weakening of their weights in these compounds). Therefore, networks of arbitrary mappings are required at the feature level to enable similarity-based matchings at the code level. High-level matching depends on a powerful machinery for low-level mapping (Prinz, 1984, 1987, 2013; Catmur et al., 2007; Heyes, 2010; Pulvermüller et al., 2014).

This raises the issue of what the functional benefits of matching may be, that is, what matching can do for cognitive systems that mapping cannot—a question reminiscent of the classical debate of the role of similarity and contiguity for the formation of associations (e.g., Warren, 1921; Turner, 1967). Matching relies on similarity or overlap, whereas mapping relies on established linkages. Matching-based systems can therefore be more flexible than mapping-based systems. They can still be functional when pre-established codes and linkages do not exist or work, for instance when novel, unfamiliar stimuli are presented or new responses are required. This seems to be the crucial asset for which cognitive systems invest substantial ressources when constructing sophisticated devices for matching. Still, we should not forget that matching at code level always requires and presupposes machinery for mapping at feature level.

## Conflict of Interest Statement

The author declares that the research was conducted in the absence of any commercial or financial relationships that could be construed as a potential conflict of interest.
